# Prosthetic Neck Fracture in Exeter V40 Stems: A Report of Two Cases and Review of Literature

**DOI:** 10.1016/j.artd.2020.11.023

**Published:** 2021-01-12

**Authors:** Balasubramanian Balakumar, Sivashanmugam Raju, Karthikeyan Chinnakkannu, Akshay Mehra

**Affiliations:** a Birmingham Orthopaedic Training Programme, Birmingham Orthopaedic Rotation, West Midlands, Birmingham; b St. Louis University School of Medicine, St. Louis, MO, USA; c Elmhurst Hospital Center/Icahn School of Medicine at Mount Sinai, New York, NY, USA; d Worcester Acute Hospital NHS Foundation Trust, UK

**Keywords:** Exeter V40 stem, Prosthetic neck fracture, Stem failure, Total hip arthroplasty, Cemented stem

## Abstract

This report contains 2 acute fractures of the Exeter V40 stem with 2 different types of neck fracture, one at the subcapital level and another at the base of the neck. A review of relevant literature provides an insight into a similar pattern of failures reported in a certain subset of cases. We hypothesize that for high-BMI cases, attention to proper preoperative templating is mandatory. The operating surgeon should try to restore offset without having to use a long head in this subgroup of patients. We feel that trying to compensate for the offset with a long head may lead to high-stress concentration in the neck. This, in turn, may be responsible for the failure of the stem, as seen in the reported cases. We advocate, based on the literature, the need to recreate the offset carefully with as big a stem as possible to avoid these unique and rare complications. Reducing such failures may further improve the overall survivorship of the V40 Exeter stem.

## Introduction

The Exeter stem is associated with a good survival outcome, even in young patients. Keeling et al. reported a 96.3% survivorship of Exeter stem at 22 years [1]. The most common causes for revision were aseptic loosening and osteolysis around the stem. Stem fracture leading to revision surgery is an infrequent complication.

Historically, the rates of stem body and neck fracture in the first-generation Exeter stem (316 L steel) were 1.87% and 3.52%, respectively [2]. Changing the polished to matt finish reduced the incidence of stem fracture to 0.2% [3] but increased the failures due to stem loosening. The Exeter stem has undergone many modifications over the years [4]. The basic geometry of the cemented Exeter stem has remained the same since 1988. The Exeter stem (Stryker Orthopedics, Mahwah, NJ) with a V40 taper was introduced in 2001. Westerman et al. have reported excellent outcomes with the V40 taper Exeter stems at 10 years. In their series, only one revision was for a stem fracture [5].

Fracture of the V40 stem is a rare complication, especially involving the neck or the junction of the neck and stem. Only a few cases have been reported to date (Table 1). We report 2 cases of such fracture, one involving the neck (trunnion) and the other involving the insertion hole, along with a review of the related literature. Both cases have been consented for publication purposes.

## Case histories

### Case 1

A 68-year-old male patient presented with sudden onset of pain in his right groin while trying to get out of his car. He was unable to weight bear on his right leg because of pain. He reported experiencing mild discomfort in the right groin for the last few months. He had undergone a right total hip replacement (Hybrid) 13 years ago.

**Figure 1:**
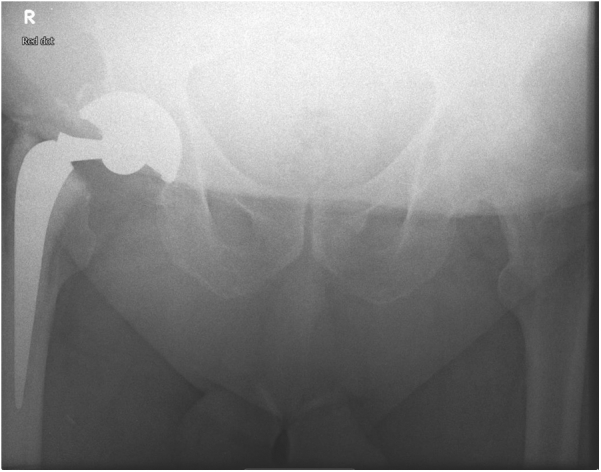
Failure at the guide hole for the insertion.

**Figure 2:**
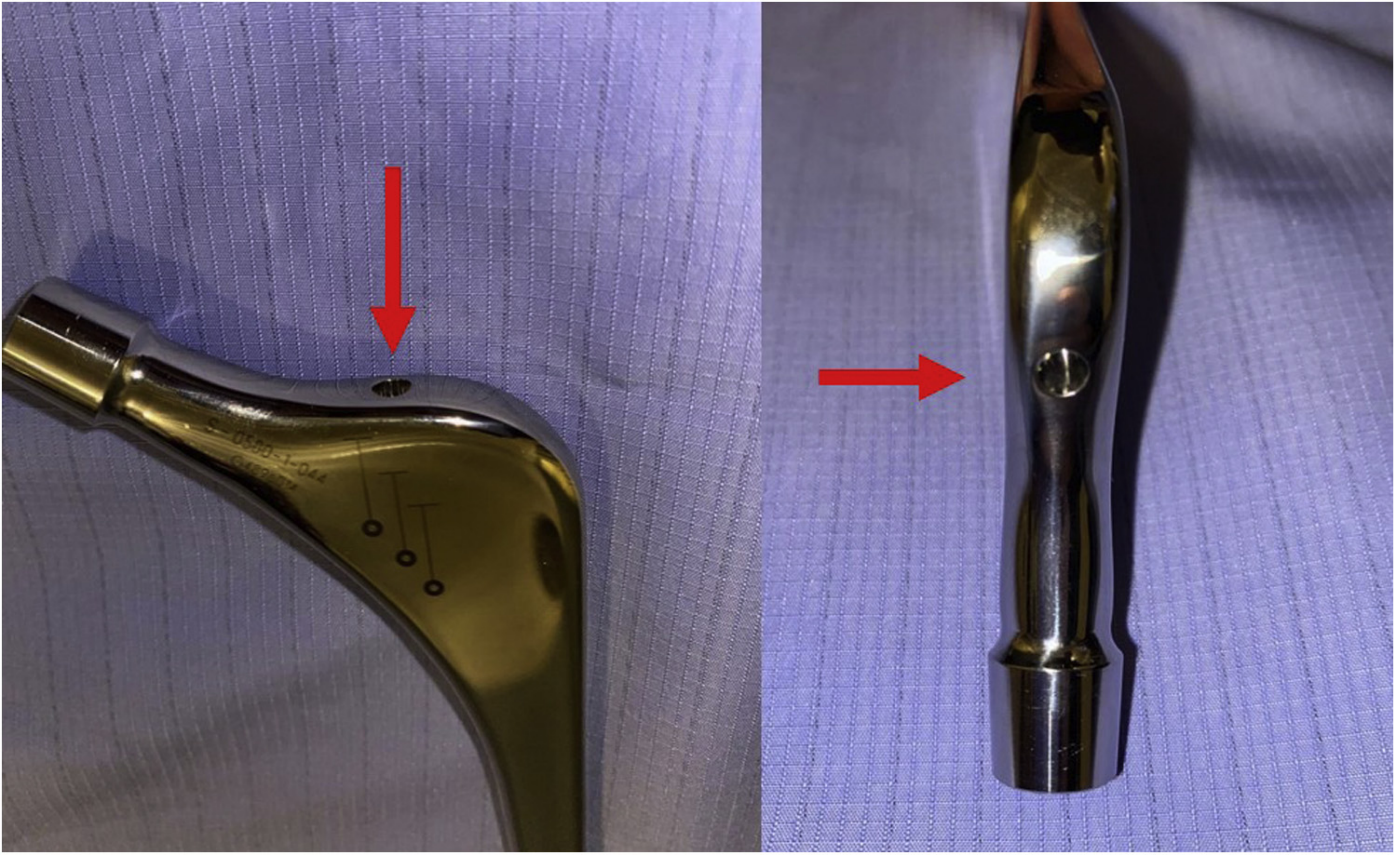
The insertion guide hole in V40 stems (red arrows).

His medical history included a high BMI of 44 (ht, 172 cm; wt, 132 kg), pleural plaques, cardiomegaly, atrial fibrillation, coronary heart disease, and venous eczema in the lower limbs. He smoked 10 cigars a day and mobilized with one walking stick. The radiographs performed showed a fracture of the Exeter stem at the level of the guide hole (Figure 1, Figure 2).

**Figure 3:**
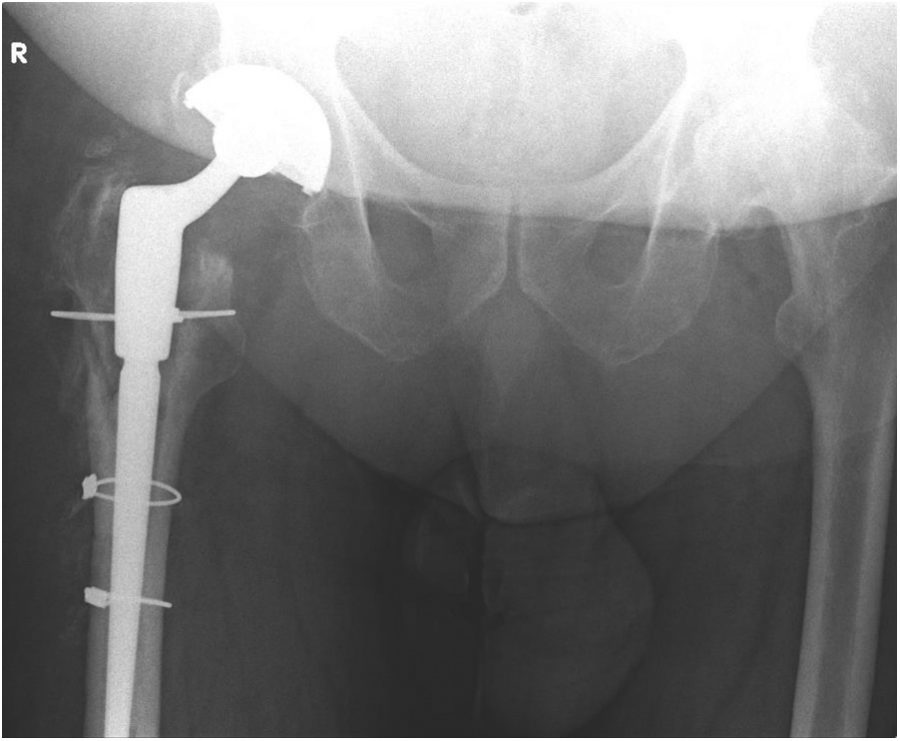
Postoperative X-ray after extended trochanteric osteotomy (ETO) and restoration stem insertion.

The primary hip was cemented Exeter V40 (44 offset number 3) stem with a 28 mm/+4 mm head and Exceed acetabular shell (64 mm CoCr) with a 28-mm ID polyethylene liner. The removal of the femoral component required an extended trochanteric osteotomy as the stem was well fixed. The cement mantle was removed using osteotomes and a high-speed burr. The revision was performed using an uncemented Stryker Restoration Modular Revision System (conical distal stem straight 17 mm × 155 mm, 25 mm/+10 mm cone body, and a V40 Vitallium 28 mm/+4 CoCr Head). The osteotomy was fixed with Dall Mile cables (Stryker Orthopedics, Mahwah, NJ). The acetabular socket was retained as it was found to be stable intraoperatively. The patient was progressing well at the 6-month follow-up (Fig. 3).

### Case 2

A 78-year-old male patient who had undergone bilateral cemented THR in 2003 presented with sudden onset of left hip pain and inability to walk after a fall in the kitchen. He lived independently and was using a walking stick/mobility scooter to get around before this fall.

His medical history included a high BMI of 50 (ht, 170 cm; wt, 145 kg), type 2 diabetes mellitus, past cerebrovascular event, hypertension, and gastroesophageal reflux disease.

**Figure 4:**
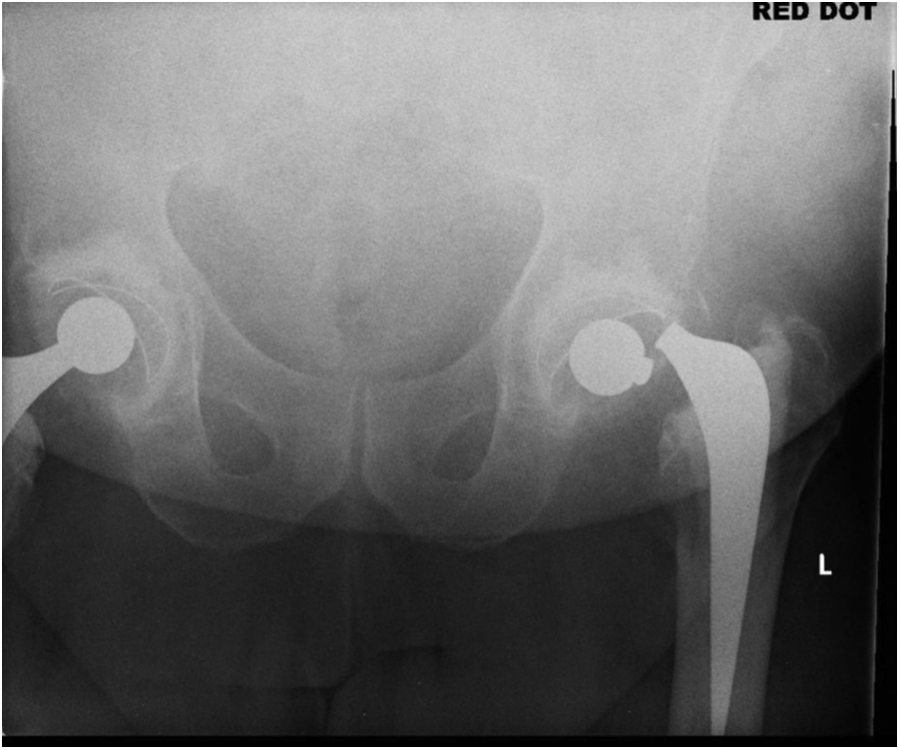
Failure at the base of trunnion.

The radiographs revealed a fracture of the Exeter stem just below the trunnion (Fig. 4).

**Figure 5:**
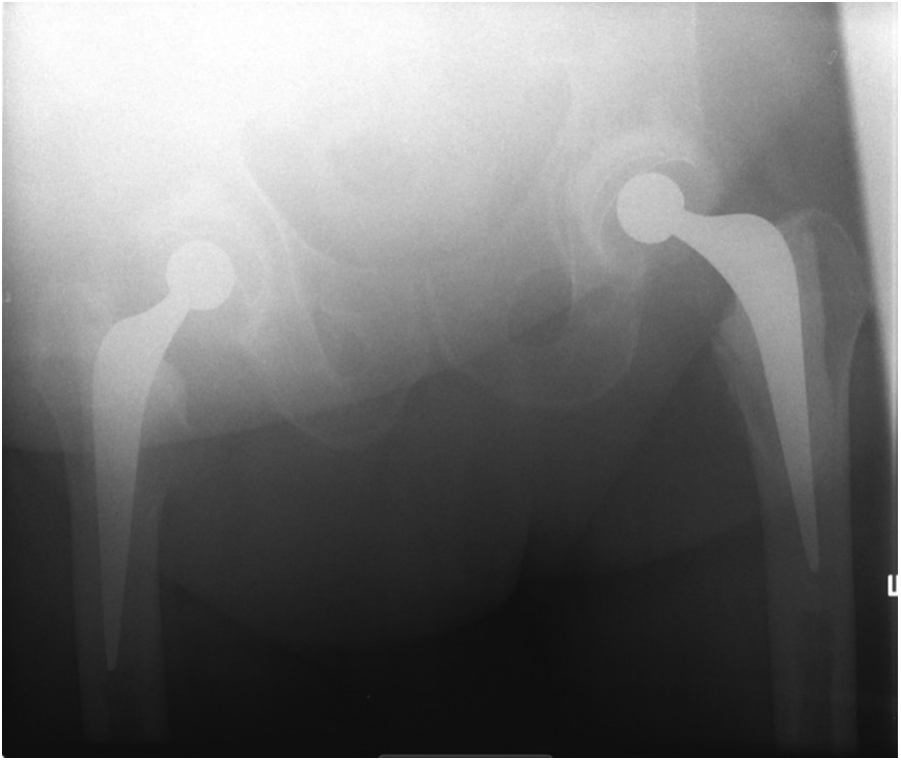
Postoperative X-ray after stem revision.

The implant used during his index procedure was Exeter V40 (44 offset no. 5) stem and a 28-mm/+4 mm head. The acetabular component was a cemented Opera cup (Smith and Nephew, Andover, MA) (53 mm OD, 28 mm ID). The acetabular cup had minimal wear and was found to be well-fixed intraoperatively. The stem was revised to a standard Exeter V40 (44 offset no. 3) stem using a cement in cement revision technique. His postoperative recovery was prolonged because of hospital-acquired pneumonia. At 6 months follow-up stage, he was ambulating with a single walking stick and progressing well (Fig. 5).

## Discussion

Despite the fact that cemented Exeter V40 stem is associated with excellent long-term survival rates [4,5], there are a few reports of stem body fracture and neck fracture. Hamlin and MacEachern reported a fracture of the stem neck at the level of the trunnion in 2014, in the Universal stem (Howmedical International Ltd., London, UK) [6]. Davies et al. first reported 4 cases of V40 stem body fractures [7]. After these initial reports, cases of neck fracture close to trunnion and insertion hole emerged, many of which were reported on the Universal Exeter stems [[8], [9], [10], [11], [12], [13]].

However, only 10 cases have been reported (Table 1) so far involving the fracture of the V40 stem [[7], [8], [9]]. The most recent version of the Exeter stem with V40 taper accounts for only 5 neck fractures. Our study attempts to bring into focus this rare complication and to highlight the risk of V40 stem fracture at the neck and insertion hole. It also reiterates the importance of considering the patients’ BMI in relation to the implants used. Both the patients referred to have high BMI with the implant head size 28/+4 mm used to restore offset and achieve the soft tissue balance. It should be noted that high BMI and the resulting stress on the implant had been suggested as the cause for the failure by Hamlin and MacEachern [6].

**Table 1:** Literature review of reported Exeter V40 stem fractures.

V40 studies	Case number	Surgery	Size	Stem fractures			Details	Possible causes
				Body	Base of neck	Subcapital neck		
Davies et al. (2013) [7]	Case 1	Primary	125	Body			CDH (125) stem 35.5, standard 26 mm FH	BMI, 44 kg/m^2^
	Case 2	Primary	150	Body			44 no. 1	BMI, 33 kg/m^2^
	Case 3		150	Body			44 no. 0, 28 standard head	BMI, 34 kg/m^2^
	Case 4	Primary	125	Body			35.5 CDH stem + mm head	BMI, 47 kg/m^2^
Garala et al. (2018) [9]	Case 1	Primary	150		Neck body junction/introducer hole		44 stem 3, 28 mm + 0 head	BMI, 37.5 kg/m^2^
	Case 2	Primary	125 Revision stem	Body			37.5, no. 0, 125 stem, 28 mm + 4	Undersized? Weight 107 kg
	Case 3	Primary	150			Subcapital	44 stem 1, +4 mm and 28 mm head	95 kg
Reito et al. (2016) [8]	Case 1	Primary				Subcapital	44 no. 3, 36 mm + 5 ceramic on ceramic	BMI, 31 kg/m^2^ well-fixed stem
	Case 2	Primary				Subcapital	44 no. 2, 40 mm + 8	BMI 27 kg/m^2^ well-fixed stem
	Case 3	Revision				Subcapital	44 no. 1, 36 + 10	BMI 38 kg/m^2^ well-fixed stem

BMI = body mass index; CDH = congenital dysplasia of hip; FH = femoral head.

Increased horizontal offset with a well-fixed stem has also been shown as a potential risk factor for fracture by Reito et al. [8]. Their series reported larger heads (36-40 mm) with more extended neck options. In our series, both the heads were 28 mm/+4 and potentially contributed to the failure similar to their cases. In the universal stems, Bolland et al. [13] had shown that 67% of the 20 fractures that occurred at the subcapital level had a plus head applied at the primary surgery. This further reiterates the inherent risk of increasing the neck length and horizontal offset.

The fracture through the neck has been noticed to occur after an acute fall akin to the fracture of a native hip neck of femur. This indicates the fatigue failure at the level of the neck due to the higher loads per cycle these implants are put through [13]. Biomechanically, the ideal THR aims to recreate the native hips offset. Failure to restore offset may lead to the inability to balance the soft tissue and gain stability. This needs accurate templating preoperatively and an attempt to achieve the same during surgery. In our cases, the surgeons had to restore the offset with +4-mm heads. Longer neck implants in high-BMI patients will result in higher stress rise due to an increase in the lever arm and thus may precipitate fatigue fracture at the weakest point. Based on the literature review, we agree, stem failure is multifactorial; however, in the cohort of V40 neck fractures reported, BMI and offset could be identified as risk factors uniformly. We propose to reduce the chances of early stem fatigue failure by restoring native offset with an appropriate stem size.

The guide hole has been alluded to as a potential weak point in the manufacture of this implant, as seen with similar fractures reported in high-BMI cases [9]. The patient with a fracture through the guide hole had reported initial prodromal pain symptoms potentially due to partial failure, which progressed soon into the catastrophic failure with an acute event. Previous retrieval studies have shown that the proximal end of the fracture through the guide hole remains smooth but gets rough as the crack progresses distally, indicating a fatigue failure pattern [9]. Also, grain size irregularity has been reported with universal stem retrieval studies by Bolland et al. [13].

In retrospect, the canal of the reported cases did look capacious, and perhaps a 50-mm offset stem would have been a better option. Given the high joint reaction forces through such hips, a high-offset stem would have the same horizontal offset achieved with a plus head on a lower offset stem. The 50-mm stems, as described by Reito et al. [8], being a monoblock metal, would have better withstood the stress than a lower offset 44 stem with plus head. We also acknowledge that the femora of such heavy patients can often be a champagne glass type and might not accept stems greater than 44 offsets.

Mild varus stem placement was noted in our second patient. It is known that a varus stem position increases horizontal offset and hence the lever arm. The Exeter stems have been shown to be tolerant of varus malposition [14,15]. We feel that in our reported cases, the cause for failure was an increased horizontal offset due to the long neck length and not the varus position.

An inadequate cement mantle may lead to stem failure at the body rather than at the neck or insertion hole [7]. The cement mantle was found to be even in both our cases with some proximal stress shielding of the calcar. We could not find any cement-related cause for the failures.

We know from the static biomechanical studies that a longer abductor lever arm reduces the joint reaction forces, but lateralization may lead to trochanteric pain and early loosening. In our cases, rather than using the 44 stem with a long neck to restore offset and achieve soft tissue balance, using a higher offset stem with a standard neck may have been a more effective solution. By doing so, the Exeter stem may get a better chance to emulate the given track record of 22.8 years as reported from the parent unit [16].

Bolland et al. [13], in 2016, reviewed all the retrieved failed Universal Exeter stems with the normal 5°40’ spigot. They found that 27 failures in their series were due to fracture at the neck level. They identified a similar subcapital pattern below the trunnion and at the base of the neck at the level of the introducer stud hole. We believe the same type of fracture results in the reported V40 stems as well.

Limitations of our interpretation based on 2 cases are well recognized. However, we could see a similar pattern happening in the literature, and this would warrant a formal registry-based assessment of all V40 stem neck fractures and possibly a biomechanical model to arrive at the safest stems to use for patients with higher BMI and narrow femoral canals. The other offshoot of this finding would be to search for sturdier implants for such heavy patients. It may be that this cohort of patients might need a shorter version (<150 cm) of high-offset stems (50 and above) to allow for this anatomical canal variation. Although V40 is currently the proven stem that is comfortably used by many surgeons who are trained in it, time may have come to reconsider the options for this category of patients given the time to failure within their lifespan.

## Summary

To conclude, we have reported yet again 2 cases of V40 stem neck fracture with a known antecedent cause, the fracture sites being subcapital at the trunnion level or base of the neck at the insertion guide hole level. These reports highlight the importance of recreating the offset with stems as much as possible to give the best possible life for the implant. V40 stems with lower offset requiring plus head to restore offset need to be cautiously considered for high-BMI cases. The jury is out for the operating surgeons to think of alternative options or some modification of the existing models for patients with high BMI and anatomical constraints.

## Conflict of interests

The authors declare there are no conflicts of interest.

## Supplementary data

Conflict of Interest Statement for Chinnakkannu

Conflict of Interest Statement for Raju

Conflict of Interest Statement for Balakumar

Conflict of Interest Statement for Mehra

## References

[1] Keeling P., Howell J.R., Kassam A.-A.M. (2020). Long-term survival of the cemented exeter universal stem in patients 50 Years and younger: an update on 130 hips. J Arthroplasty.

[2] Fowler J.L., Gie G.A., Lee A.J., Ling R.S. (1988). Experience with the Exeter total hip replacement since 1970. Orthop Clin North Am.

[3] Gie G.A., Ling R.S., Timperley A.J. (1996). Stem fracture with the Exeter prosthesis. Acta Orthop Scand.

[4] Exeter hip System:34. http://www.rogerbrighton.com/pdf/exeter-technical-guide.pdf.

[5] Westerman R.W., Whitehouse S.L., Hubble M.J.W., Timperley A.J., Howell J.R., Wilson M.J. (2018). The Exeter V40 cemented femoral component at a minimum 10-year follow-up: the first 540 cases. Bone Joint J.

[6] Hamlin K., MacEachern C.F. (2014). Fracture of an exeter stem: a case report. JBJS Case Connect.

[7] Davies B., Branford White H., Temple A. (2013). A series of four fractured Exeter^TM^ stems in hip arthroplasty. Ann R Coll Surg Engl.

[8] Reito A., Eskelinen A., Pajamäki J., Puolakka T. (2016). Neck fracture of the Exeter stem in 3 patients. Acta Orthop.

[9] Garala K., Laios T., Lawrence T. (2018). A report of 3 cases of Exeter V40 Stem fracture and explanation of possible causes. Hip Int.

[10] Facek M., Khatib Y., Swarts E. (2016). Prosthetic fracture of a cemented exeter femoral stem (case report). Reconstruct Rev.

[11] Moloney D.P., Hurley R.J., Harty J., Guerin S. (2019). History, treatment and analysis of a rare form of Exeter stem fracture. BMJ Case Rep.

[12] O’Neill G.K., Maheshwari R., Willis C., Meek D., Patil S. (2011). Fracture of an Exeter ‘cement in cement’ revision stem: a case report. Hip Int.

[13] Bolland B.J.R.F., Wilson M.J., Howell J.R., Hubble M.J.W., Timperley A.J., Gie G.A. (2017). An analysis of reported cases of fracture of the universal exeter femoral stem prosthesis. J Arthroplasty.

[14] Hossain M., Thomas G., Beard D., Murray D., Andrew G. (2012). The consequences of varus implantation of a taper-slip cemented femoral stem. Orthop Proc.

[15] Takada R., Whitehouse S., Hubble M. (2019). Does varus or valgus alignment of the exeter stem influence survival or patient outcome in total hip arthroplasty? a review of 4126 cases with a minimum follow-up of five years. Orthop Proc.

[16] Petheram T.G., Whitehouse S.L., Kazi H.A. (2016). The Exeter Universal cemented femoral stem at 20 to 25 years: a report of 382 hips. Bone Joint J.

